# Induction of altered states of consciousness during Floatation-REST is associated with the dissolution of body boundaries and the distortion of subjective time

**DOI:** 10.1038/s41598-024-59642-y

**Published:** 2024-04-23

**Authors:** Helena Hruby, Stefan Schmidt, Justin S. Feinstein, Marc Wittmann

**Affiliations:** 1 Institute for Frontier Areas of Psychology and Mental Health, Freiburg, Germany; 2 Department of Psychosomatic Medicine and Psychotherapy, Medical Center - University of Freiburg, Faculty of Medicine, University of Freiburg, Breisgau, Germany; 3 Float Research Collective, Kihei, HI USA

**Keywords:** Altered states of consciousness, Body boundaries, Time perception, Relaxation, Floatation-REST, Psychology, Health care

## Abstract

Floatation-REST (Reduced Environmental Stimulation Therapy) minimizes stimulation of the nervous system by immersing subjects in an environment without sound or light while they effortlessly float in thermoneutral water supersaturated with Epsom salt. Here we investigated the relationship between altered states of consciousness (ASC) and its association with the affective changes induced by Floatation-REST. Using a within-subject crossover design, 50 healthy subjects were randomized to 60 min of Floatation-REST or 60 min of Bed-REST (an active control condition that entailed lying supine on a warm waterbed in a dark and quiet room). Following Floatation-REST, subjects felt significantly more relaxed, less anxious, and less tired than after Bed-REST. Floatation-REST also induced significantly more pronounced ASC characterized by the dissolution of body boundaries and the distortion of subjective time. The loss of body boundaries mediated the loss of anxiety, revealing a novel mechanism by which Floatation-REST exerts its anxiolytic effect.

## Introduction

Altered states of consciousness (ASC) induced through different psychological and pharmacological methods are characterized by a variety of short-term phenomenal effects on the perception of the internal (feelings, thoughts) and external world (hallucinations)^1^. Personal reports and empirical studies have indicated that ASCs are characterized by changes in the senses of self and time, peak states often described as ego dissolution and a reduction of the sense of time^2–4^.

Systematic studies with psychedelic compounds, such as psilocybin, have shown that profound alterations in time perception are accompanied by a reduced sense of the bodily self, including a feeling of unity with the world^5^. Highly different time experiences may occur during different phases of psychedelic states, but peak experiences are typically reported to be those of timelessness and selflessness, as experienced during the intake of psychedelics such as ayahuasca or psilocybin^6,7^. Experienced meditators typically report that the meditation session felt much shorter than it actually lasted in clock time^8^. In one quantitative study, experienced meditators subjectively perceived their body boundaries less strongly, they paid less attention to time, and felt time pass more quickly compared to the reading condition (control condition), in which the subjects read a text while adopting the same body posture as in the meditation session^9^. The joint modulation of the experience of time and self is also found with other ASC induction methods like the Ganzfeld technique of perceptual deprivation caused by exposure to an unstructured, uniform visual and auditory perceptual field (e.g., a red light and white noise)^10,11^, and when drinking red wine^12^. Similarly, the effects of imagery and suggestion in hypnotized individuals leads to changes in time perception, i.e. the underestimation of duration, and lower self-awareness^13,14^.

The above-mentioned studies demonstrate that variations in states of consciousness are often defined by the up- and down-modulation of (bodily) self-consciousness and subjective time. An intensified awareness of the self (the body, emotional feelings) correlates with an intensified awareness of time as exemplified by the boredom often experienced while waiting for something to happen^15^. A decreased awareness of the self is accompanied by a decreased awareness of time, such as during flow states when absorbed in an exciting activity like when playing a video game in virtual reality^16^. On a neural level, this relationship between subjective time and the bodily self is related to activity in the insular cortex, which plays a fundamental role in the perception of time passage and the estimation of duration^17^. Thus, one’s sense of time may be intricately related to emotional and visceral processes that share a common neurobiological substrate within the insular cortex^18^.

Many psychiatric and neurological syndromes are characterized by distortions in emotional and physical self-regulation, as well as distortions in time perception^19^. For example, patients suffering from depression and anxiety report a subjective slowing down of time and being ‘stuck in time’^20,21^. The core features of timelessness and selflessness are the opposite of psychiatric conditions in which patients suffer from a hyperawareness of time and the self^22^. The positive effects of psychedelic treatment^23^, physical exercise and meditation^24^, as well as exposure to Floatation-REST^25^ (Reduced Environmental Stimulation Therapy) in patients with psychiatric syndromes may be due to the transitory downregulation of the experience of self and time, e.g., a decrease in self-related thoughts and in attending less to the passage of time.

Floatation-REST is a technique in which one can almost weightlessly float on the surface of supersaturated water heated to skin temperature. This is done in a special floating facility (a tank, a pool) that is isolated from external stimuli, i.e. people typically float in a quiet and dark environment, which typically leads to deep physical and mental relaxation^26^. Prior research investigating Floatation-REST has found largely salubrious effects on both physical and mental health^27^ (participants with pathologically defined stress-related symptoms) including the reduction of stress in healthy participants^28^, pain^29,30^ (participants with stress related ailments and muscle pain), and anxiety^25,31^ (participants across the spectrum of anxiety and stress-related disorders and patients with generalized anxiety disorder, GAD). However, little is known about how the float environment can induce ASC. Seminal work showing how this technique leads to more relaxation, improved mood, and sometimes strong altered states of consciousness has been performed by a group of researchers at Karlstad University in Sweden over the last two decades^32–34^. This research found that Floatation-REST did induce ASC, including out-of-body experiences and altered time perception. Al Zoubi and colleagues^35^ showed how functional connectivity between the posterior default-mode network (DMN) and the posterior insula and somatosensory cortices was significantly reduced following exposure to Floatation-REST. These two networks are related to the narrative and bodily self, respectively, and the posterior insula specifically to the experience of the passage of time^17,18^.

Prior research from our laboratory has utilized a validated battery of questions to assess the changes in the sense of time and self in relation to mood in various states. Among others, the state questionnaires encompassed those as used in relaxation music therapy^36^, exposure to silence in nature^37^, meditation in very experienced meditators^9^ and in meditation-naïve individuals^38^, the multi-modal Ganzfeld^10^, and when individuals reported on their last sexual orgasm^39^. We also used the scales when people had to stay in a waiting room or in virtual reality without distraction^40^. Based on the real waiting room, we designed a waiting room in virtual reality, which was experienced through a VR headset. Moreover, to assess states of flow, we used the scales after participants had played a dynamic video game in virtual reality^16^.

**Figure 1:**
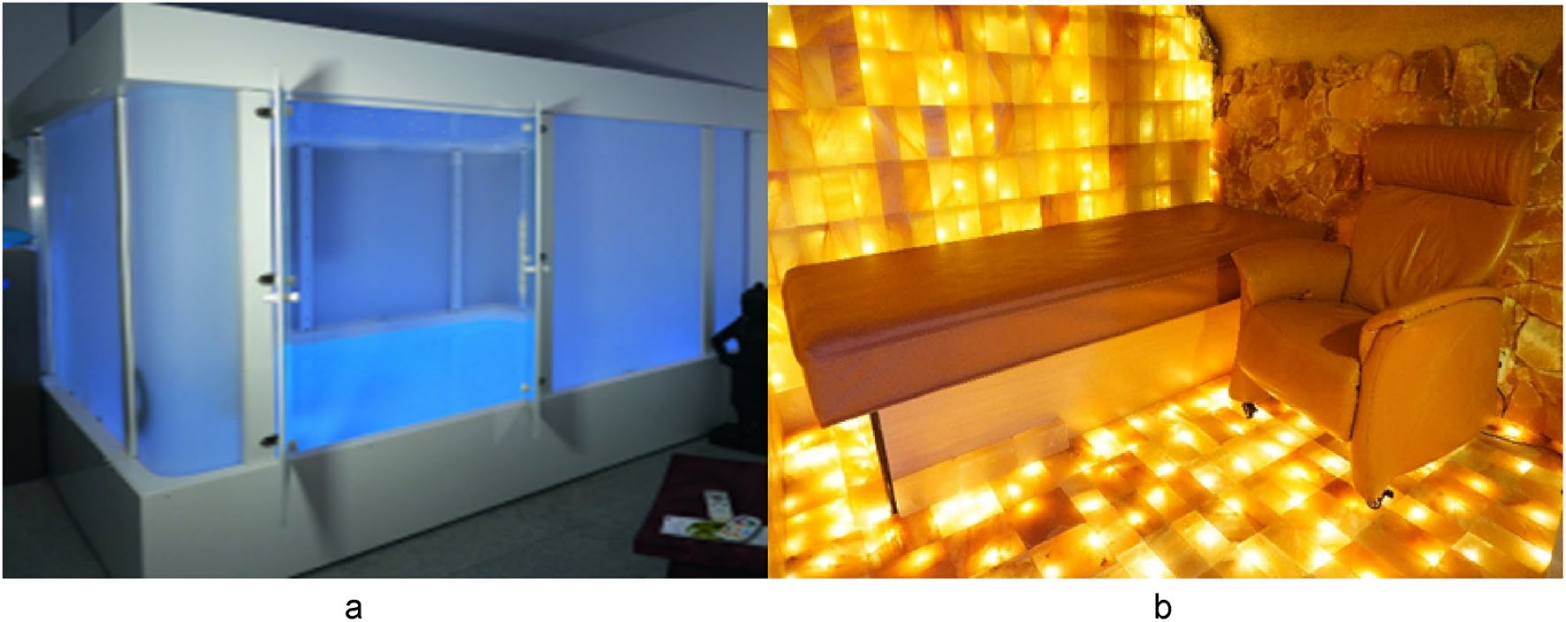
Two photos depicting the Floatation-REST cabin (**a**) and the Bed-REST environment (**b**). Note that the two REST environments were experienced in complete darkness by the participants. Photos: © Tommi Nübling: https://www.wake.de/

In the present study, we used these measures pertaining to the sense of time and bodily self to assess these dimensions of ASC during Floatation-REST. We applied a within-subjects crossover design and examined ASC in 50 healthy participants who underwent 60 min of Floatation-REST or a control condition (Bed-REST), where they lay supine on a waterbed in a dark, soundproof room for the same amount of time. The control condition chosen was not unlike that of the "Chamber-REST" condition used by Suedfeld^41^. According to Suedfeld, the test subjects in the REST condition usually lie on their backs on a bed or sit in a chair. Our more comfortable waterbed condition comes even closer to the experience of Floatation-REST. This is an active control condition, where many features are the same as in Floatation-REST, i.e. where all external sights and sounds are minimized while the subject lies in a relaxed supine position on a bed filled with warm water. ASC were assessed using the Phenomenology of Consciousness Inventory (PCI; Pekala^42^), the Perceived Body Boundaries Scale (PBBS; Dambrun^43^), and visual analog scales assessing different facets of the sense of time Jokic et al.^44^. We also measured changes in levels of emotional well-being, anxiety, stress, relaxation, and fatigue (emotional state variables) (Fig. 1).

**Figure 2:**
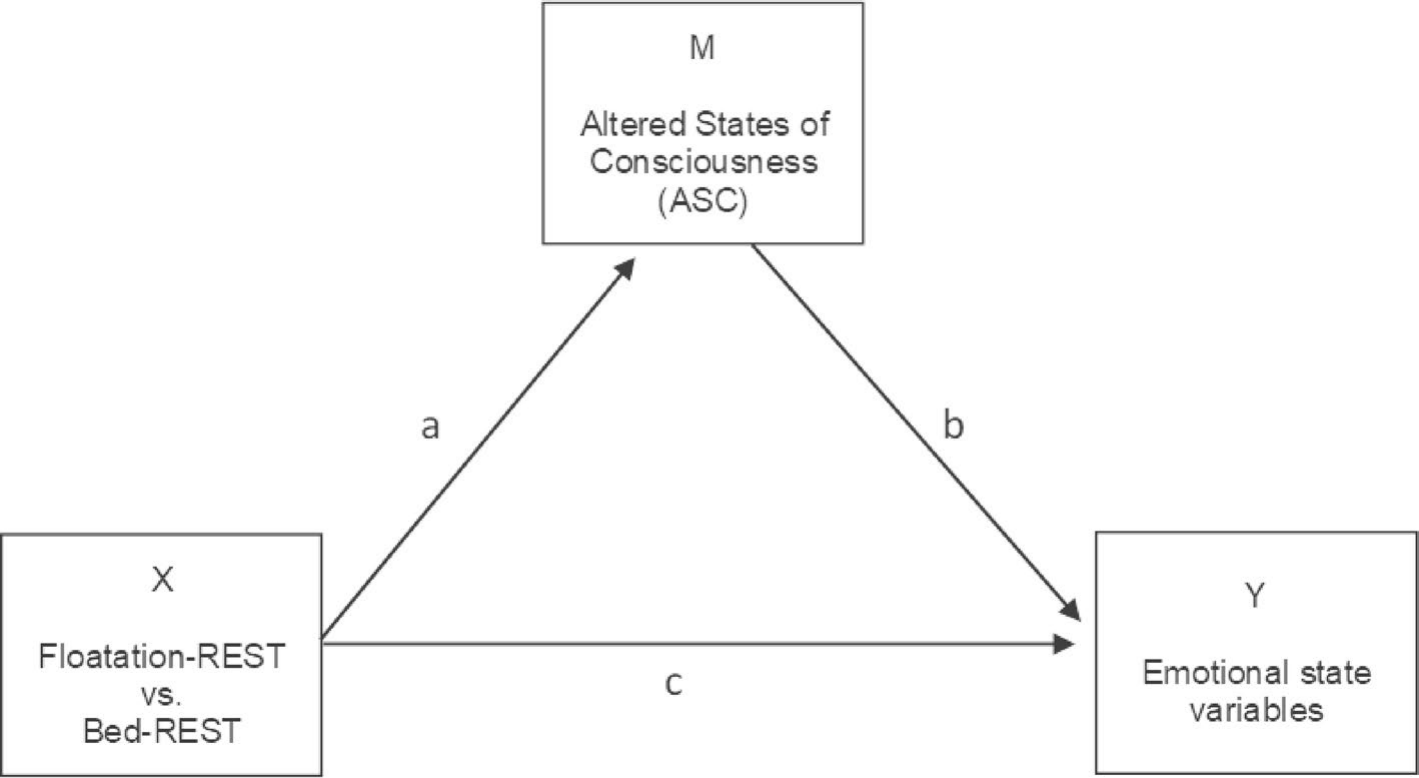
Diagrammatic representation of the hypothesized effects in a mediation analysis. There are potential direct (**c**) and indirect effects (**a**, **b**) of the condition (Floatation-REST vs. Bed-REST; independent variable X) on emotional state variables (dependent variable Y) as potentially mediated (M) through ASC (time perception, body boundaries, altered states dimensions in PCI).

Based on the aforementioned previous research on the positive effects of Floatation-REST on mood and the induction of ASC, this exploratory study tested two main hypotheses: (1) Floatation-REST, as compared to Bed-REST, will lead to greater improvements in emotional well-being (less anxiety, tension, and stress, and more relaxation), and (2) Floatation-REST, as compared to Bed-REST, will lead to ASC including a greater loss of body boundaries and a greater distortion in subjective time. With a mediation analysis we test whether the direct effects of Floatation-REST vs. Bed-REST on emotional well-being are partially or completely mediated by the experienced ASC (see Fig. 2). In addition, we assessed the personality trait of mental absorption to investigate how individual personality differences account for the effects found on ASC. A prior meditation study demonstrated that the ability to become absorbed in certain situations in daily life has an influence on the depth of meditation; the more pronounced the ability to absorb, the deeper the reported meditative states^45^. Here we predicted that higher levels of absorption would be related to higher levels of ASC induced during Floatation-REST.

## Methods

### Participant recruitment

We aimed at a total of 50 participants. The convenience sample of 25 male and 25 female participants was recruited through word of mouth and through a leaflet, as participants recommended the study to other individuals. Despite uncertainties in the specific experimental conditions (no prior knowledge of effect sizes) and considering multiple statistical tests for several dependent variables, a sample size of N = 50 was chosen. With an effect size of 0.6, and a power of 0.80, 38 participants are required to achieve a p-value of 0.01 in paired two-tailed Wilcoxon tests (36 for paired *t* tests). One study using a depth-relaxation method with participants inexperienced in meditation^36^ showed a Cohen's *d* value of 0.62 in the relaxation question used here, that is, between the depth-relaxation intervention and a control condition. This is a conservative estimate since we expected stronger effects between Floatation-REST and Bed-REST.

Demographic information was collected (age, sex, education level), and inclusion and exclusion criteria were reviewed during a phone screening. Inclusion criteria were age (18–49), very good oral and written German language skills, and physical and mental health. Exclusion criteria were acute or chronic physical illness (e.g. incontinence, cold symptoms), acute or chronic psychiatric or neurological disorders (e.g. depression, epilepsy, attention deficit hyperactivity disorder), medication that impairs physical or mental functioning and the autonomic nervous system, addiction disorders, open or bleeding wounds, as well as contraindications due to a medically diagnosed illness, pregnancy, and menstruation at the time of the floating session. The participants' prior experiences with floating and other contemplative practices were also recorded. The exclusion criteria were asked again using a checklist before each REST session.

Participants came three times to the study site. The first session was a familiarization session where subjects had the opportunity to try out both REST conditions for 30 min each. This was done to reduce the effects of novelty and ensure that any questions could be answered prior to commencing the following two 60-min REST sessions. The participants were randomized into either the experimental condition (60 min of Floatation-REST) or the control condition (60 min of Bed-REST) in the two subsequent sessions following the familiarization session. The order was balanced across all three sessions to control for sequence effects (the order within the familiarization session, and the order of the two measurement conditions). The time interval between sessions 1 and 2 as well as between sessions 2 and 3 was typically one week.

The study was pre-registered at the University Clinic of Freiburg (https://www.uniklinik-freiburg.de/zks.html; FRKS004085) and it was approved by the Clinical Trials Register (DRKS) as the German WHO primary registry. The clinical trial registration number is DRKS00033671 and the date of registration is: 20.02.2024. The study was designed in accordance with the principles of the Declaration of Helsinki and was approved by the Ethics Committee of the University Freiburg. We confirm that all research was performed in accordance with relevant guidelines and regulations. All participants were informed about the confidentiality and anonymity of the data in a participant information sheet and gave written informed consent. Participation in the study was voluntary, and a session could be terminated at any time. Each participant received a compensation of ten euros per session (a sum of 30€). The study was financed by a Hanns Seidel Foundation scholarship for Helena Hruby from funds of the Federal Ministry of Education and Research (BMBF), Germany.

## Trait measures

### Absorption (TAS)

The German version^46^ of the TAS (Tellegen Absorption Scale^47^) consists of 34 items with answer categories on a 5-point Likert scale (0–4) with answer categories applicable/slightly/partly/mostly/absolutely applicable. Example items include: "I like to watch how clouds change their shape" (item 6), "My thoughts often appear as images rather than words" (item 22), and "I think that different smells have different colors" (item 33). Absorption is defined as ‘‘…a disposition for having episodes of ‘total’ attention that fully engage one’s representational (i.e., perceptual, enactive, imaginative, and ideational) resources.”^40^. The internal consistency value is a Cronbach's alpha of 0.89^46^. The German norm data comparable to our sample in age has an average score of 60.05 with a standard deviation of 19.98^46^. Our sample on average has a value of 71.6 (S.D.: 22.4).

### Perceived stress (PSQ)

The Perceived Stress Questionnaire (PSQ) by Levenstein et al.^48^ measures retrospective individual stress perception during the preceding two years. The questionnaire was validated on a German sample^49^ and comprises 20 items that are assigned to seven scales in the German version. Participants can indicate whether the statements in the items apply "almost never", "sometimes", "often", or "mostly" on a 4-point Likert scale of 1–4. The internal consistency value is a Cronbach's alpha of 0.86^49^. The German norm data for our sample have a stress score of 34% (S.D.: 16%) from the possible maximum score^49^. Our sample on average has a stress value of 34.33% (S.D.: 13.4%) from the possible maximum score.

## State measures

The state measures were filled out either before and after the REST conditions (pre-post-measurements) or only after (post-measurement).

### Emotional reaction (SAM, pre-post-measurements)

The Self-Assessment-Manikin Scales (SAM^50^) measures both (1) positive and negative valence and (2) the current arousal level with five figures, the Manikins, by answering the two questions: "How positive/negative do you feel right now?" and "How aroused/calm do you feel right now?".

### State anxiety (STAI, pre-post-measurements)

The German short version of the State-Trait-Anxiety Inventory (STAI) by Spielberger^51^ contains ten items measuring subjectively experienced current anxiety. Participants answer how well the following descriptions of emotions apply to themselves in the present moment: "How much do the following descriptions of emotions apply to you right now?" on an 8-point Likert scale. An overall score with a minimum of 10 and a maximum of 80 points is calculated. The internal consistency of the State-Anxiety Scale has a Cronbach’s alpha of 0.90^52^.

### Tension and stress level (VAS, pre-post-measurements)

Participants rated their individual relaxation and stress levels on a 100-mm visual analog scale (VAS) by making a vertical line. The relaxation question asked, "How relaxed do you feel right now?" (endpoints: "extremely relaxed" (0), "extremely tense (100)") and the stress question asked, "How high do you estimate your current stress level?" (endpoints: "extremely low", "extremely high").

### Fatigue (VAS, pre-post measurement)

Level of fatigue before the intervention was indicated on a 100-mm VAS that asked, "How sleepy do you feel?" (endpoints: "extremely awake: 0"; "extremely sleepy: 100"). Additionally, we asked participants after the session how sleepy they felt during the REST conditions: "How sleepy did you feel?" with the same answer endpoints of “awake” to “extremely sleepy” on a VAS.

### Mind-wandering (VAS, post-measurement)

Mind wandering is assessed after the intervention using a 100-mm VAS by asking the question, "How many thoughts are currently going through your head?" (endpoints: "none at all", "extremely many").

### Subjective time perception (STSS, post-measurement)

The Inventory on Subjective Time, Self, Space (STSS^44^) assesses subjective time perception by using mostly 100-mm VAS. The scales consist of the following questions, which are answered as spontaneously as possible: "How often did you think about time during REST?" (Thinking about time: endpoints: "not at all" to "extremely often"); "How fast did time seem to pass for you during REST?" (Subjective passage of time: endpoints: "extremely slow" to "extremely fast"). "Did you lose your sense of time during REST?" (Time distortion: endpoints: "not at all" to "extremely strong”). The question "How long did the 60-min REST session subjectively feel?" assesses the subjectively felt duration of the exposure to the intervention. Subjects indicated their sense of duration by writing the respective number of minutes into a field.

### Body boundaries (PBBS, post-measurement)

Changes in perceived body boundaries during the intervention were assessed using the Perceived Body Boundaries Scale (PBBS^43^). Participants can choose between the drawings of seven human bodies with different levels of visible body boundaries. 1 represents a weak body boundary, and 7 represents a strong body boundary.

### General change in states of consciousness (PCI, post-measurement)

The German version of the Phenomenology of Consciousness Inventory (PCI)^42^ consists of 53 items that can be answered on a 7-point Likert scale ranging from 0 to 6. The following 12 dimensions are given: self-awareness (D15), altered state (D16), internal dialogue (D17), rationality (D18), voluntary control (D19), memory (D20), arousal (D21), positive (D22) and negative affect (D23), altered experience (D24), imagination (D25), and attention (D26). The PCI is a valid measurement instrument for the assessment of subjective phenomenological experiences in different contexts (e.g., hypnosis, relaxation, meditation, and Ganzfeld)^1^.

### Floatation-REST

The float tank, a “Cabin for two” built by Floataway (Norfolk, United Kingdom), is located in the Prana Health Practice in Freiburg, Germany (see Fig. 1a). The room contains the float tank (width 180 cm, length 237 cm, height 225 cm) and a shower. The pool was filled with 1100 L of water mixed with 550 kg of Epsom salt (magnesium sulfate) creating a water height level of 27 cm. The water and air temperature was matched to human skin temperature (~ 35 °C) and the air temperature at the rim of the pool was around 33.4 °C, slightly lower than the water temperature based on the relative humidity in the air (84%). The water pH values ranged from slightly acidic to neutral between 6.8 and 7.2 (total alkalinity: 150 ppm). The density of the saltwater was maintained at a specific gravity of around 1.25 for all float sessions, allowing for effortless floating with about 1/3 of the body above the water surface. The float tank was placed on a bed of butyl rubber floor springs, effectively isolating the pool from the building and preventing all structure-borne noises from entering the water. The entire floating room had non-slip flooring and handholds for getting in and out of the tank. The room was illuminated while the participant changed and showered. The external lighting in the room was switched off by the experimenter only after the subject had entered the pool. The subject switched off the pool light using an air switch. The blue LED light inside the float cabin could be switched on again (in case someone felt uncomfortable) and an alarm button could be pressed to trigger an audible alarm signal in case of an emergency. The exact instruction the experimenter gave was: *You will now float for 60 min. Please lie on your back in the middle of the pool and do nothing else. Please try not to fall asleep during this time, as measurements are taken after the resting phase. After the 60 min have elapsed, a gong will sound which signals the end of the session and the light will turn on again.*

#### Bed-REST

In the control condition, participants were situated in a separate, quiet room where they lied supine on a 90 × 220 cm waterbed (Classic Wasserbett SOLO) built by Aqua Comfort GmbH (Paderborn, Germany) while wearing clothing (see Fig. 1b). The waterbed has a calming level of 90%, meaning that the post-swing time of water movement when you lie down in the bed is maximum 2 s. The surface temperature of the imitation leather cover was held constant across subjects at 28 °C, which is the manufacturer's recommended comfort temperature. When the lights were turned off by the experimenter, the room was completely dark and a low salt content could be perceived in the air due to salt stones that were placed around the perimeter of the room. The average room temperature was ~ 23 °C. Participants were instructed to do nothing and lie on their backs for 60 min without falling asleep. The exact instruction the experimenter gave was: *You will now rest for 60 min. Please lie on your back and do nothing else. Please try not to fall asleep, as measurements will be taken after the resting phase. After the 60 min have elapsed, I will turn on the light and open the door.*

#### Procedures

At the beginning of the first session (familiarization session), participants received information about the experiment and signed an informed consent. Then they filled out questionnaires related to their trait characteristics (TAS, PSQ). The subjects were exposed to 30 min of Floatation-REST and Bed-REST (balanced succession across participants) in randomized order. Comprehensive written and verbal information instructions were given regarding the exact procedure and all relevant safety aspects before each REST session.

Participants were requested to shower and wash their hair with the provided shampoo before entering the pool naked in the Floatation-REST condition. They were reminded to remove or store all valuable items, such as jewelry, cell phone, hair clips, makeup, and clothing, and to plug their ears with earplugs while they were still dry to provide better acoustic isolation and protect from salt crystals becoming lodged in the ear canal (of note, the same earplugs were also used during the Bed-REST condition). Urgent needs, such as using the restroom, were taken care of before the REST conditions. The room was lit when the participants entered the float cabin; the ceiling light was turned off by the experimenter once the participant had entered the tank. The participants stepped into the pool using the handles and laid down on their backs in the middle of the pool. The participants were instructed to turn off the pool light when they were in their preferred resting position. The participant indicated through an intercom when he/she turned off the light so that the experimenter could accurately time the floatation session. The end of the session was indicated when the pool light went on and a gong sounded. The participants left the pool to shower and dress. To indicate the end of the 60 min session in the Bed-REST condition the experimenter opened the door of the salt room and switched on the light. The post-condition questionnaires were completed in a nearby room on a table (of note, the pre-condition questionnaires were completed in the same room). Participants were briefly asked if any adverse experiences had occurred before they filled out the questionnaires.

The two 60-min REST sessions took place after the familiarization session. Either the Floatation-REST condition was completed first followed by the Bed-REST condition or vice versa. In both cases, participants filled out state-related questionnaires during the pre-measurement, followed by the 60-min REST intervention, and then the completion of the post-measurement questionnaires. Overall, all three sessions lasted a maximum of two hours each.

#### Statistical analysis

The main intention of this study was to determine the differences in experience between Floatation-REST and Bed-REST. Statistical calculations were conducted using SPSS (Version 24). After testing for normal distribution (Kolmogorov–Smirnov and Shapiro Wilk), it was found that 28 according to the Kolmogorov–Smirnov-test and 30 according to the Shapiro Wilk test out of a total of 62 variables were not normally distributed (2 trait variables, 30 variables in the Floatation-REST condition, and 30 variables in the Bed-REST condition). Therefore, non-parametric testing was applied, and the Wilcoxon test for dependent samples was used to calculate the difference between the Floatation-REST and Bed-REST conditions, as well as the post- vs. pre-intervention effects. Median and interquartile ranges are indicated. The difference score between the post- (t2) and pre-measurement (t1) was used in the calculations (DIFF: t2-t1) for variables collected before and after the REST interventions.

The association between indices of ASC and emotional states was calculated using Spearman correlations. To determine whether the influence of the two conditions on state variables was mediated by ASC (X = state variables, Y = conditions, M = ASC; Fig. 2), we performed a mediation analysis with the PROCESS Macro (Version 3.4.1) by Hayes^53^. The influence of trait-related variables on state-related variables was determined using regression analysis with bootstrapping procedures that allows for the use of data that is not normally distributed^54^. False Discovery Rate (FDR^55^) calculations were used to correct the significance level for multiple comparisons.

## Results

### Sample characteristics

In total, 53 subjects were recruited. Two subjects reported falling asleep during the Bed-REST condition. In the Floatation-REST condition, one subject did not turn off the light as she felt uncomfortable in the dark. These three subjects were therefore excluded. The remaining 50 participants (25 males and 25 females) had an average age of 29.5 years (SD = 6.2), and most achieved a high level of education (68% had a university degree, 20% had a high-school diploma, and 12% had a middle-school diploma). 90% of participants had never floated before, 6% reporting that they floated once before, and 2% reporting that they floated twice before (of note, one subject failed to answer this question). 10% of the participants practiced contemplative techniques daily (e.g., yoga, meditation, chi-gong, etc.), 38% on a weekly basis, 22% practiced monthly, 10% reported having hardly any contemplative experiences (i.e., yearly or only a few times in their life), and 12% reported having no contemplative experiences. None of the 50 participants terminated the Floatation-REST or Bed-REST session prematurely or withdrew from the study. All participants successfully completed both sessions, remaining in the dark for the full 60 min during each session. Regarding potential sex differences across all variables (FDR adjusted for n = 44 calculations), women were more stressed before Floatation-REST than men (*p* = 0.001), women were significantly more tired during Floatation-REST than men (*p* = 0.001), and women significantly were in a higher positive mood (SAM valence) after vs. before Bed-REST.

### Post- versus pre-intervention effects on mental states

After the Floatation-REST condition (Table 1, see Supplementary Fig. S1), participants reported significantly lower levels of arousal (*p* < 0.0001) and anxiety (*p* < 0.0001), they were less tense (*p* < 0.0001), and had less stress (*p* < 0.0001) compared to before. They also had greater positive affect (SAM valence; *p* = 0.017). After the Bed-REST condition (Table 1, see Supplementary Fig. S2), participants reported significantly lower levels of arousal (*p* < 0.0001) and anxiety (*p* < 0.0001), they were more relaxed (*p* = 0.0001), felt less stressed (*p* < 0.0001), and were more tired (*p* < 0.0001). Importantly, when comparing the levels of fatigue prior to entering the two interventions (median fatigue: Floatation-REST = 53; Bed-REST = 51), there was no significant difference (*p* = 0.992).

**Table 1:** Pre- to Post-REST differences in mental states during Floatation-REST and Bed-REST.

Scale [range]	Floatation-REST median [Mean*] (Interquartile)		Wilcoxon	Bed-REST median [Mean*] (Interquartile)		Wilcoxon
			Test			Test
	Pre	Post	p	Pre	Post	p
SAM-Valence [-2 …. 2]	1 [0.62] (1)	1 [0.92] (1)	**0.017**^**a**^	1 [0.68] (1)	1 [0.80] (1)	0.413
SAM-Arousal [1 …. 5]	3 (2)	2 (1)	**0.0001**^**a**^	3 (1)	2 (1)	**0.0001**^**a**^
Anxiety (STAI) [10 …. 80]	39 (16)	25 (12)	**0.0001**^**a**^	34 (13)	27 (10)	**0.0001**^**a**^
Tension (VAS) [0 …. 100]	54 (26)	15 (13)	**0.0001**^**a**^	44.5 (31)	20 (19)	**0.0001**^**a**^
Stress (VAS) [0 …. 100]	51 (32)	19 (20)	**0.0001**^**a**^	46 (33)	21 (23)	**0.0001**^**a**^

Wilcoxon test for pre-post measurements within each condition. ^a^Meets FDR-adjusted significance of *p* < 0.05 for 22 statistical tests (Tables 1, 2, 3). * The mean values of a variable are additionally shown when the medians do not exhibit the significant differences between pre- and post-REST. Next to visual analogue scales (VAS), the Self-Assessment-Manikin (SAM) scales with the subscales valence and arousal as well as the State-Trait-Anxiety Inventory (STAI) were used for the assessment of mental states.

Significant values are in bold.

### Floatation-REST versus Bed-REST on mental states

When comparing the two conditions (Table 2, see Supplementary Fig. S3), Floatation-REST led to significantly more relaxation (*p* = 0.009) and less fatigue (*p* = 0.018) as well as to greater anxiety reduction (*p* = 0.033) as compared to Bed-REST. Regarding time perception, Floatation-REST resulted in a stronger loss of the sense of time (*p* = 0.007), on average longer and more accurate estimates of the duration of the intervention (*p* = 0.012), and a stronger dissolution of bodily boundaries (*p* < 0.0001) as compared to Bed-REST (Table 3, see Supplementary Fig. S4). Participants reported higher values in altered states (*p* = 0.003) and altered experiences (*p* = 0.005) during Floatation-REST than during Bed-REST as measured with the PCI (see Supplementary Table S1). Importantly, the mean value of duration estimates after Floatation-REST does not adequately represent participants’ experience. A bimodal distribution in duration estimates (Supplementary Fig. S8) is visible, with many individuals having smaller and many other individuals larger estimates than 60 min. This result mirrors the stronger loss of the sense of time after floating.

**Table 2:** Differences in mental states between Floatation-REST and Bed-REST.

Scale [range]	Floatation-REST median [Mean*] (Interquartile)	Bed-REST median [Mean*] (Interquartile)	Wilcoxon test
			p
DIFF SAM-Valence [− 2 …. 2]	0 [0.30] (1)	0 [0.12] (2)	0.339
DIFF SAM-Arousal [1 …. 5]	− 1 [− 0.86] (2)	− 1 [− 0.71] (1)	0.520
DIFF Anxiety (STAI) [10 …. 80]	− 10 [− 11.66] (11)	− 10 [− 7.94] (14)	**0.033**^**a**^
DIFF Tension (VAS) [0 …. 100]	− 35 (24)	− 20.5 (34)	**0.009**^**a**^
DIFF Stress (VAS) [0 …. 100]	− 30 (27)	− 18.5 (26)	0.125
Fatigue during REST (VAS) [0 …. 100]	54 (30)	65 (35)	**0.010**^**a**^
Mind wandering post- REST (VAS) [0 …. 100]	33.5 (30)	31.5 (35)	0.605

Wilcoxon Test for the difference between medians of pre-post measurements of the respective condition (t2–t1 = DIFF) or Wilcoxon Test for post-measurements. Measurements related to the state after Floatation- and Bed-REST. ^a^Meets FDR-adjusted significance of *p* < 0.05 for 22 statistical tests (Tables 1, 2, 3). * The mean values of a variable are additionally shown when the medians do not exhibit the significant differences between the REST conditions. Next to visual analogue scales (VAS), the Self-Assessment-Manikin (SAM) scales with the subscales valence and arousal as well as the State-Trait-Anxiety Inventory (STAI) were used for the assessment of mental states.

Significant values are in bold.

**Table 3:** Difference between Floatation-REST and Bed-REST regarding time and bodily-self perception.

	Floatation-REST Median (Interquartile)	Bed-REST Median (Interquartile)	Wilcoxon test
			p
Thinking about time (VAS) [0 …. 100]	35 (29)	28.5 (31)	0.274
Subjective passage of time (VAS) [0 …. 100]	49.5 (38)	58 (20)	0.127
Time distortion (VAS) [0 …. 100]	70 (28)	58 (47)	**0.007**^**a**^
Estimation of duration (VAS) [0 …. 100]	60 (36.25)	49 (20)	**0.012**^**a**^
Body boundaries (PBBS) [1 …. 7]	3.5	5	**0.0001**^**a**^
	2	2	

Wilcoxon Test with indication of medians/interquartiles. Measurements related to the time during Floatation and Bed-REST. ^a^Meets FDR-adjusted significance of *p* < 0.05 for 22 statistical tests (Tables 1, 2, 3). Next to visual analogue scales (VAS) the Perceived Body Boundaries Scale (PBBS) was used for the assessment of the sense of time and self.

Significant values are in bold.

We correlated the variables `time distortion’ and `body boundaries’, which are relevant for measuring ASC, with the PCI variables `altered experience’ (PCI D24) and `altered state’ (PCI D16) (Table 4). The Spearman correlation coefficients showed an association between time distortion and ‘altered experience’ (r = 0.326, *p* = 0.021). Body boundaries showed highly significant correlations with both ‘altered experience’ (r = − 0.419, *p* = 0.002) and `altered state’ (r = − 0.417, *p* = 0.003). There was no significant correlation between time distortion and body boundaries (r = − 0.070, *p* = 0.630).

**Table 4:** Correlation of the ASC variables.

		Altered experience (PCI D24) [	Altered state (PCI D16)
		0 …. 6]	[0…. 6]
Time distortion (VAS)	*r*	0.326	0.231
[0 …. 100]	*p*	**0.021**^**a**^	0.106
Body boundaries (PBBS)	*R*	− 0.419^a^	− 0.417
[1 …. 7]	*p*	**0.002**^**a**^	**0.003**^**a**^

^a^Meets FDR-adjusted significance of *p* < 0.05 for 5 statistical tests. Two sub-dimensions of the Phenomenology of Consciousness Inventory (PCI) were correlated with the visual analogue scale (VAS) of time distortion and the Perceived Body Boundaries Scale (PBBS).

Significant values are in bold.

### Correlations between ASC and emotional-state variables

We conducted Spearman correlations within the Floatation-REST condition between the emotion-related variables (SAM-Arousal, stress level, anxiety, and tension which were reduced after Floatation-REST (see Table 1) and the ASC variables (loss of sense of time, loss of body boundaries, altered experience, altered state), which were increased during Floatation-REST vs. Bed-REST (Table 3, Supplementary Table S1). The correlation between the subjective stress level and altered experiences (PCI) during Floatation-REST was significant (r = − 0.447, *p* = 0.001). The more altered experiences that occurred during floating, the lower the stress level after Floatation-REST (see Supplementary Table S2, Fig. S7).

### Mediation analyses

**Figure 3:**
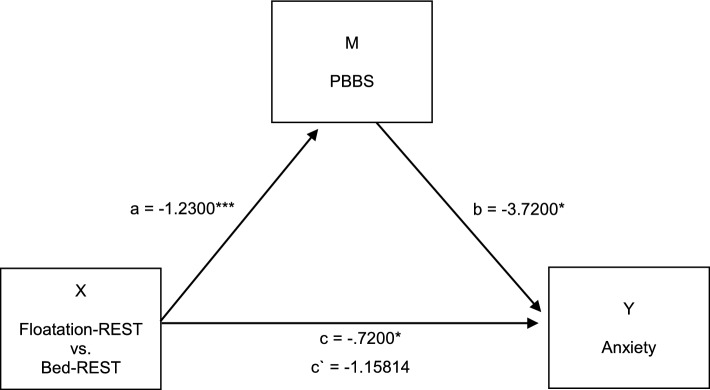
Mediation analyses: body boundaries as mediators M mediate the effects of condition (X) on anxiety (Y) completely. Significance levels: *p < 0.05; **p < 0.01, ***p < 0.001. PBBS = Perceived Body Boundaries Scale.

In the mediation analyses we only selected variables that showed a significant difference between Floatation-REST and Bed-REST, with the mediators M (loss of sense of time, loss of body boundaries, and the PCI dimensions of `altered state’ / `altered experience’) and the Y variables state anxiety reduction and tension (Tables 2, 3; Supplementary Table S1). Only the mediation analysis with body boundaries as the mediator M and state anxiety as the Y variable was significant (Fig. 3). Body boundaries completely mediated the effect of the experimental conditions on state anxiety reduction; c = − 3.72, *p* = 0.017; CI [− 6.74; − 6.95], c’ = − 1.58, *p* = 0.349; CI [− 0.4.95; 1.79]. Floatation-REST leads to a stronger dissolution of body boundaries compared to Bed-REST (a = − 1.23; *p* = 0.0001; CI [− 0.79; − 0.66]). The weaker the body boundaries, the lower the state anxiety level after floating (b = − 3.72, *p* = 0.0170; CI [− 6.74; − 0.69]).

### Influence of trait absorption on state variables

While all dependent state variables were considered when there were significant differences between the two REST conditions after FDR adjustment (tension, fatigue, loss of sense of time, subjective duration, body boundaries, PCI: altered state/experience), only the body boundaries (as a dependent variable; DV) was found to be affected by trait absorption as an independent variable as demonstrated by a non-parametric regression analysis (employing a bootstrapping procedure; with non-standardized coefficients). The stronger the absorption capacity, the more the body boundaries dissolved, r = − 0.018, *p* = 0.016, [− 0.032; − 0.002] (Supplementary Table S3).

## Discussion

This study explored the relationship between ASC and the affective changes induced during REST in a sample of 50 healthy subjects. Across both conditions (60 min exposure to Floatation-REST and 60 min exposure to Bed-REST), the subjective experience of arousal, anxiety, tension, and stress significantly decreased after REST. However, Floatation-REST led to a significantly stronger reduction in anxiety and tension as compared to Bed-REST, and participants also reported feeling more fatigued during Bed-REST. Since we used a within-subjects crossover design, these significant between-condition differences demonstrate that Floatation-REST was inherently more relaxing and refreshing than Bed-REST. This corroborates prior float studies utilizing other control conditions including a zero-gravity chair^35^, a relaxing film^56^, and a dry-REST condition^57,58^.

We deliberately chose one of the strongest known control conditions to test for the specific effects of Floatation-REST. The Bed-REST condition had a similar level of sensory reduction for sight and sound, and a similar body position, with participants lying supine on top of a warm cushion of water (~ 28 °C). In addition, participants were exposed to both conditions for an equivalent amount of time and were given the same instruction set. Thus, the immersion into water heated to skin temperature (~ 35 °C) and saturated with Epsom salt during Floatation-REST constituted the decisive difference between the REST conditions. The other main difference was air temperature, which was also matched to skin temperature during Floatation-REST whereas during Bed-REST it was kept at typical room temperature (~ 23 °C). Thus, during Floatation-REST, the close matching of the ambient temperatures to that of the skin appears to blur the boundaries between air, body, and water. This blurring of boundaries leads to an ASC where one can no longer discern where their body begins and ends, the essence of body dissolution. A dissolution of body boundaries as measured with the specific PBBS (Perceived Body Boundaries) scale has been shown in body scan meditation in meditation-naïve^43^ as well as experienced meditators^9^. Together with our Floatation-REST results with the PBBS as yet another positive induction method for relative body dissolution, a systematic review pertaining to the dynamics and variability in perceived selfhood should be undertaken as a future endeavor^59^.

As a consequence of the tight temperature calibration within the float environment, along with the reduced proprioceptive input stemming from effortlessly floating in supersaturated saltwater, we found that body dissolution was significantly higher during Floatation-REST as compared to Bed-REST. The experienced loss of body boundaries and the significant distortion of time during Floatation-REST, experiences which correlated with the two ASC dimensions of the PCI (Supplementary Table S1), fit conceptual ideas concerning the embodiment of time. Depending on the alterations in consciousness, the body and time senses can both be up- and down-regulated^2,3^ (see introduction). Especially in certain peak experiences of altered states such as in meditation or under the influence of psychedelic substances, time and self are experienced as radically changed^6,9^. Qualitative studies point to disruptions in the sense of time in a majority of individuals exposed to Floatation-REST^32,60^. Although subjects reported a disruption of subjective time during Floatation-REST, they nevertheless on average overestimated the duration of the floating session relative to the waterbed session (in fact they were on average accurate relative to clock time). However, a plot of the histogram of participants’ duration judgments complements the finding of a disruption of subjective time during Floatation-REST. Although the 50 participants on average were more accurate, a bimodal distribution is visible with many individuals having clearly smaller and many clearly larger estimates than 60 min after floating (see Supplementary Fig. S8). In the waterbed session subjects on average judged the duration with a modus of 50 min within a normal distribution and not as many clearly deviating estimates (see Supplementary Fig. S9).

Aside from the changes in subjective self and time, which are hallmarks of ASC^2,3^, according to the PCI participants reported significantly stronger ASC on the two sub-dimensions ‘altered state’ and ‘altered experience’. The ‘altered state’ subscale determines whether the state of consciousness deviates fundamentally from normal waking consciousness (e.g., “I felt an extremely different and unusual state of consciousness”). The ‘altered experience’ subscale captures different aspects of ASC more precisely. These include changes in body and time perception, changes in perceptual experience, as well as spiritual and transcendent experiences. For example, one item says, “I experienced intense unity with the world; the boundaries between me and the environment dissolved away”; another is formulated as, “Existence became deeply sacred and meaningful”. Regarding correlations between the ASC variables and the other mental states during Floatation-REST, only one correlation was significant after adjusting for multiple comparisons: the more altered the experience (PCI subscale D24), the greater the reduction in reported stress.

We conducted mediation analyses to investigate whether the stronger ASC in Floatation-REST constituted a mediation effect on the other mental states or whether the differences between the intervention types demonstrated a direct effect on these emotional states. We found one significant mediation effect: the loss of body boundaries was responsible for mediating the higher degree of anxiety reduction found during Floatation-REST. As compared to Bed-REST, Floatation-REST led to a stronger dissolution of body boundaries, and the dissolution of body boundaries in turn led to lower levels of state anxiety after the float session, highlighting a novel mechanism by which floating may exert its anxiolytic effect.

We also examined the potential effects of individual differences on subjective experience using regression analyses: A stronger absorption capacity leads to a stronger dissolution of body boundaries. Absorption can be defined as the ability to ‘totally’ direct attention to one's activities, such as listening to music, watching a movie, or engaging in a conversation^47^. In our case, participants with a greater absorption capacity were able to experience the dissolution of the body boundaries more strongly. Being absorbed as an experienced state is a typical feature of ASC, trait absorption being the ability to enter such absorbed states more easily^61^. Our study thus corroborates findings of a study with experienced meditators where individuals who had a stronger propensity of absorption in daily life reported deeper meditative states^45^.

### Limitations and future directions

Although the control condition was chosen to mimic Floatation-REST, there were additional differences between the two conditions. While participants were lying on the waterbed wearing comfortable clothing, no clothes were worn during floating, and wearing clothes may make it more difficult to dissolve bodily boundaries. The room temperature in the Bed-REST condition also did not match the water temperature of 35 °C. These factors may have influenced the dissolution of bodily boundaries. Another specific aspect concerns our sample. A majority of the participants had contemplative experiences on a daily (10%), weekly (38%), and monthly (22%) basis. This could have had a positive impact on the experience of ASC, as contemplative practices facilitate easier access to ASC^9,43^. One further limitation is we only employed a single 1-h REST session for each condition, following the initial 30 min familiarization session, and it is unknown whether additional sessions of longer duration would lead to more pronounced ASC ASC. We could have included a further, simpler control condition, i.e. a regular bed in a silent and dark room, in order to assess whether the waterbed was indeed such a strong active control condition. It is also worth discussing whether unnoticed lighter forms of sleeping could have occurred in both conditions. We excluded three participants who reported to have fallen asleep for an extended period of time. It is likely that some further participants during the one-hour phase were transiently in drowsy states, more so in the Bed-REST condition, as on average participants reported greater fatigue afterward as compared to Floatation-REST. To not interfere with the immersive experience we could, however, not monitor participants with EEG measurements or behavioral tasks, e.g., through the intercom system.

Our study demonstrates that Floatation-REST is a safe and effective means to induce certain facets of ASC among healthy individuals. It leads to a dissolution of perceived bodily boundaries and altered time perception. The mediation analysis revealed that the dissolution of bodily boundaries can be considered a key factor in reducing state anxiety. This encourages further investigations into the psychotherapeutic effects of ASC in future Floatation-REST studies in order to examine whether ASC also mediates the reduction in state anxiety found in clinically anxious patients^25^. Likewise, it is possible that body dissolution could be underlying the post-float improvement in body image recently found in patients with anorexia nervosa^62^. Future float studies testing clinical populations will need to assess whether the induction of ASC plays a mediating role in Floatation-REST’s beneficial effects on mental health.

Given the limitations of current treatment approaches for depression and anxiety disorders, there is currently a great need for and interest in complementary therapeutic approaches. For example, the application of psychedelics as an additional treatment option for patients with anxiety and depressive disorders is being researched and has had positive preliminary results^63,64^. A randomized controlled clinical study (Phase II) is currently investigating the effects of psilocybin in 144 patients with severe, treatment-resistant forms of depression^65^. Floatation-REST could present an attractive non-pharmacological alternative for inducing ASC with the advantage of causing no or minimal side effects^34,62,66,67^ making it a safe and relaxing method to induce ASC and explore the structure and processes of time-consciousness and self-consciousness, which were both strongly altered in our study.

### Supplementary Information

Supplementary Information.

## Data Availability

The experimental data that support the findings of this study are available via OSF repository at: https://osf.io/5rzbv/.
