# Supplementary material for: Induction of altered states of consciousness during Floatation-REST is associated with the dissolution of body boundaries and the distortion of subjective time
Source: Sci Rep. 2024 Apr 23;14:9316. doi: 10.1038/s41598-024-59642-y (PMC11039655; doi:10.1038/s41598-024-59642-y)
Supplement: Supplementary file 1 — Supplementary Information. [file 41598_2024_59642_MOESM1_ESM.docx]

Induction of altered states of consciousness during Floatation-REST is associated with the dissolution of body boundaries and the distortion of subjective time

Helena Hruby^1,2*^, Stefan Schmidt^2^, Justin S. Feinstein^3^, Marc Wittmann^1*^

^1^ Institute for Frontier Areas of Psychology and Mental Health, Freiburg, Germany, ^2^ Department of Psychosomatic Medicine and Psychotherapy, Medical Center - University of Freiburg, Faculty of Medicine, University of Freiburg, Germany, ^3^ Float Research Collective, Kihei, HI, United States.

*hruby@igpp.de; [*wittmann@igpp.de](mailto:*wittmann@igpp.de)

This document contains additional Figures and Tables to the manuscript: **Induction of altered states of consciousness during Floatation-REST is associated with the dissolution of body boundaries and the distortion of subjective time**. The raw data and the codes for analysis can be accessed from https://osf.xxx. Contact Hruby (hruby@igpp.de) or Marc Wittmann (wittmann@igpp.de) for more information or questions about the manuscript.


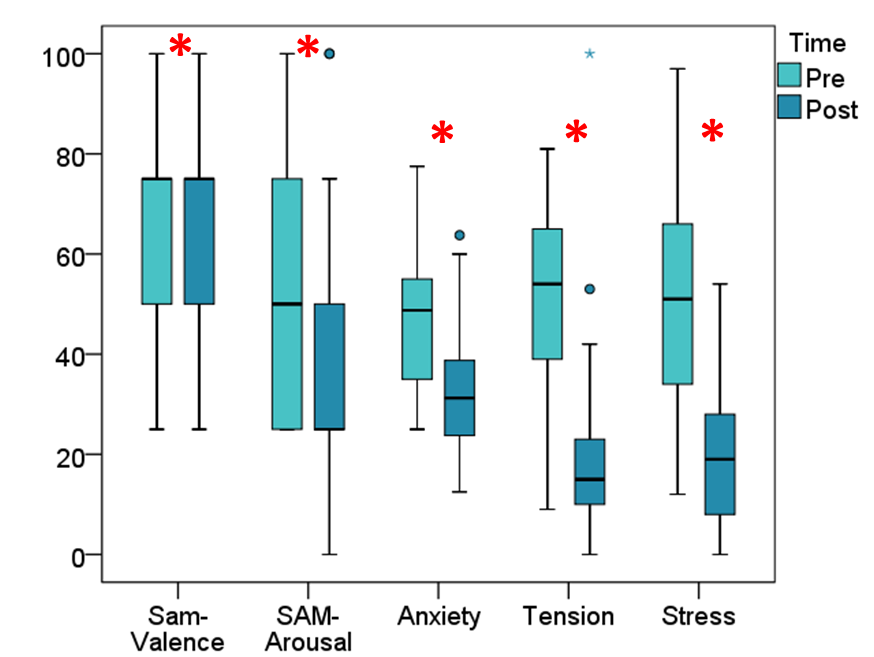


**Figure S1 Differences in mental states within the Floatation-REST condition.** Boxplots for the pre-measurement (light blue) and post-measurement (dark blue) of the variables SAM-Valence, SAM-Arousal, Anxiety, Tension and Stress. The x-axis represents the time of measurement (Pre and Post). The Boxplot show the median (line that divides the box into two parts), the interquartile range (range between the 25th and 75th percentile, IQR), the lower first quartile (25% of scores fall below) and the upper third quartile (75% of scores fall below), the minimum and maximum scores (shown at the end of the whiskers) plus outliers (data point outside the whiskers, 25th and 75th percentile ± 1.5 * IQR, respectively). As the variables had different scales we standardized the variables SAM-Valence, SAM-Arousal and Anxiety to percent values adjusted to the largest value per scale. Significant differences are indicated with large red asterisks.

**
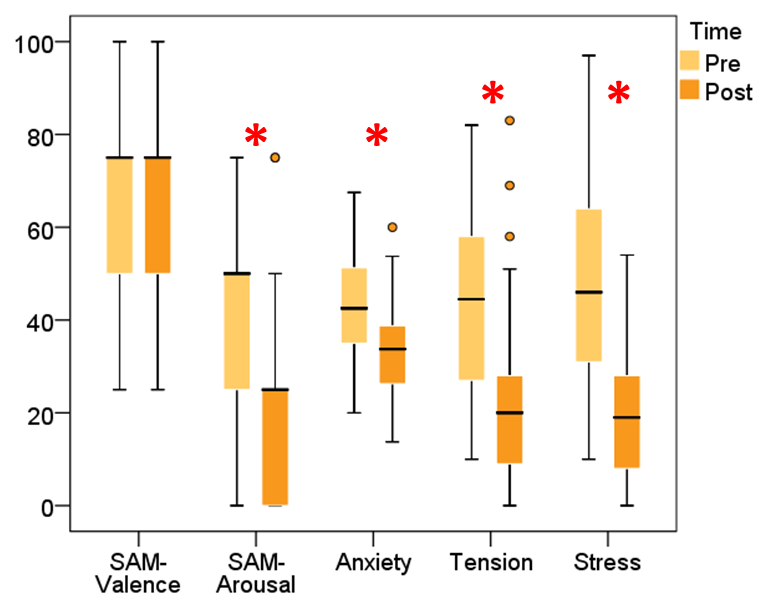
**

**Figure S2 Differences in mental states within the Bed-REST condition.** Boxplots for the pre-measurement (yellow) and post-measurement (orange) of the variables SAM-Valence, SAM-Arousal, Anxiety, Tension and Stress. The x-axis represents the time of measurement (Pre and Post). The Boxplot show the median (line that divides the box into two parts), the interquartile range (range between the 25^th^ and 75^th^ percentile, IQR), the lower first quartile (25% of scores fall below) and the upper third quartile (75% of scores fall below), the minimum and maximum scores (shown at the end of the whiskers) plus outliers (data point outside the whiskers, 25^th^ and 75^th^ percentile **±** 1.5 * IQR, respectively). As the variables had different scales we standardized the variables SAM-Valence, SAM-Arousal and Anxiety to percent values adjusted to the largest value per scale. Significant differences are indicated with large red asterisks.


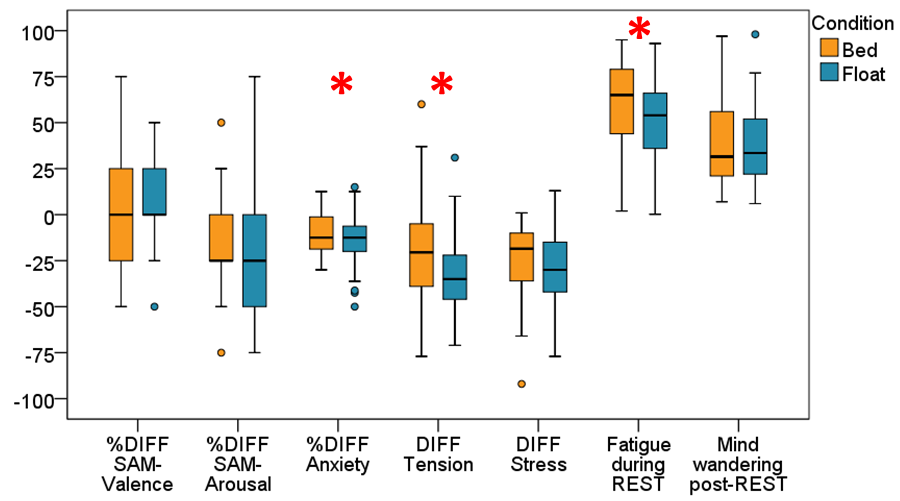


**Figure S3 Differences in mental states between Floatation-REST and Bed-REST.** Boxplots for the following variables are broken down by condition (orange = Bed; blue = Float): SAM-Valence, SAM-Arousal, Anxiety, Tension and Stress. For variables with a pre (t1)- and post (t2)-measurement, the difference between t2 and t1 was formed (t2-t1 = DIFF). The x-axis represents the condition (Bed and Float). The Boxplot show the median (line that divides the box into two parts), the interquartile range (range between the 25^th^ and 75^th^ percentile, IQR), the lower first quartile (25% of scores fall below) and the upper third quartile (75% of scores fall below), the minimum and maximum scores (shown at the end of the whiskers) plus outliers (data point outside the whiskers, 25th and 75th percentile ± 1.5 * IQR, respectively). As the variables had different scales we standardized the variables SAM-Valence, SAM-Arousal and Anxiety to percent values adjusted to the largest value per scale. Significant differences are indicated with large red asterisks.


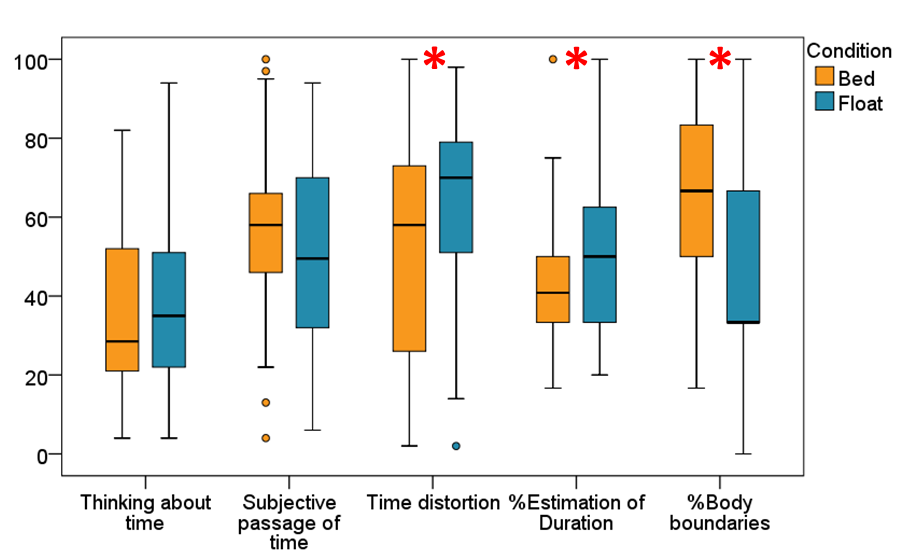


**Figure S4 Differences between Floatation-REST and Bed-REST regarding time and bodily-self perception**. Boxplots for the following variables are broken down by condition (orange = Bed; blue = Float): Thinking about time, Subjective passage of time, Time distortion, Estimation of duration, Body boundaries. The x-axis represents the condition (Bed and Float). The Boxplot show the median (line that divides the box into two parts), the interquartile range (range between the 25^th^ and 75^th^ percentile, IQR), the lower first quartile (25% of scores fall below) and the upper third quartile (75% of scores fall below), the minimum and maximum scores (shown at the end of the whiskers) plus outliers (data point outside the whiskers, 25th and 75th percentile ± 1.5 * IQR, respectively). As the variables had different scales we standardized the variables Estimation of Duration and Body boundaries to percent values adjusted to the largest value per scale. Significant differences are indicated with large red asterisks.

**Table S1. Differences between Floatation-REST and Bed-REST regarding the Phenomenology of Consciousness Inventory (PCI) variables**

|  | **Floatation-REST**  Median  (Interquartile) | **Bed-REST**  Median  (Interquartile) | **Wilcoxon**  **Test**  p |
| --- | --- | --- | --- |
| Self-awareness (D15)  [0 …. 6] | 4  (2) | 4.000  (1.333) | 0.407 |
| Altered state (D16)  [0 …. 6] | 3.333  (2.333) | 2.000  (1.667) | **0.003^a^** |
| Internal dialogue (D17)  [0 …. 6] | 3.000  (3.000) | 3.000  (3.000) | 0.553 |
| Rationality (D18)  [0 …. 6] | 3.667  (1.667) | 3.667  (1.667) | 0.528 |
| Volitional control (D19)  [0 …. 6] | 2.667  (1.333) | 2.833  (1.667) | 0.784 |
| Memory (D20)  [0 …. 6] | 4.167  (2.000) | 3.833  (2.333) | 0.237 |
| Arousal (D21)  [0 …. 6] | 1.500  (1.500) | 1.500  (1.500) | 0.462 |
| Positive affect (D22)  [0 …. 6] | 2.083  (1.500) | 1.500  (2.000) | **0.020** |
| Negative Affect (D23)  [0 …. 6] | 0.833  (1.167) | 0.583  (1.167) | 0.663 |
| Altered Experience (D24)  [0 …. 6] | 2.538  (1.462) | 1.846  (1.615) | **0.005^a^** |
| Imagery (D25)  [0 …. 6] | 3.000  (1.750) | 3.250  (1.750) | 0.321 |
| Attention (D26)  [0 …. 6] | 4.000  (1.000) | 4.000  (1.200) | 0.893 |

Wilcoxon Test with indication of medians/interquartiles. Measurements related to the time during Floatation- and Bed-REST. ^a^ Meets FDR-adjusted significance of p < 0.05 for 12 statistical tests (12 PCI dimensions).

**Figure S5:** The scatterplot shows the correlation between the variables “Altered experience” (PCI, D24) on the x-axis and “Body boundaries” (PBBS) on the y-axis.

**Figure S6:** The scatterplot shows the correlation between the variables “Altered state” (PCI, D16) on the x-axis and “Body boundaries” (PBBS) on the y-axis.

**Figure S7:** The scatterplot shows the correlation between the variables “Altered experience” (PCI, D24) on the x-axis and “Stress level” (VAS) on the y-axis.

**Table S2. Floatation-REST: correlation of emotional-state variables with ASC**

|  |  | Time Distortion  [1 …. 100] | Body Boundaries (PBBS)  [1 …. 7] | Altered Experience  (PCI D24)  [0 …. 6] | Altered State  (PCI D16)  [0…. 6] |
| --- | --- | --- | --- | --- | --- |
| DIFF  SAM-Arousal  [1 …. 5] | *r*  *p* | -0.005  0.973 | **0.282**  **0.047** | -0.224  0.117 | -0.223  0.120 |
| DIFF Stress level (VAS)  [0 …. 100] | *r*  *p* | -0.083  0.566 | **0.299**  **0.035** | **-0.447^a^**  **0.001** | **-0.291**  **0.040** |
| DIFF  Anxiety (STAI)  [1 …. 8] | *r*  *p* | -0.036  0.805 | 0.228  0.111 | -0.217  0.131 | -0.279  0.050 |
| DIFF Tension (VAS)  [0 …. 100] | *r*  *p* | 0.001  0.993 | 0.094  0.517 | -0.203  0.156 | -0.259  0.069 |

DIFF = Difference between pre- and post-measurements. ^a^ Meets FDR-adjusted significance of p < 0.05 for 16 statistical tests

**Table S3. Regression analysis of trait absorption on *body boundaries***

| *DV: Body Boundaries (PBBS)* | Regression  Coefficient ^b^ | Standard Error ^b^ | p | 95% Confidence Interval  Lower Limit Upper Limit |
| --- | --- | --- | --- | --- |
| Trait absorption | **-0.018*** | 0.007 | 0.016 | -0.032 -0.002 |

Significant coefficients: * p < 0.05; ** p < 0.01, *** p < 0.001. ^b^ Confidence intervals and standard errors via BCa (bias-corrected and accelerated) bootstrapping with 1000 BCa samples. Note that the regression coefficients are not standardized values.


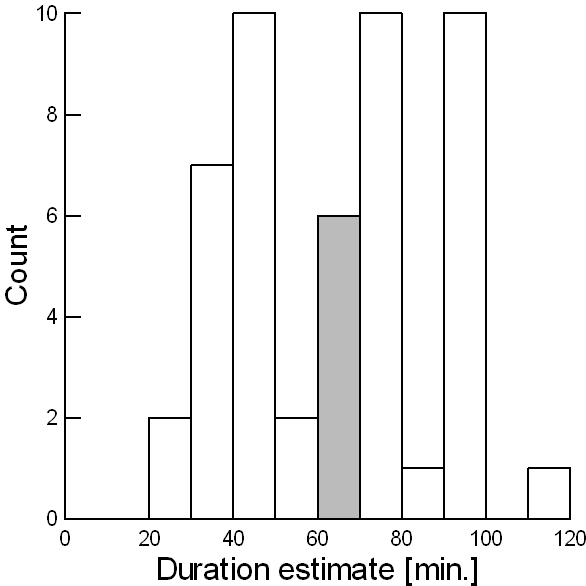


**Figure S8:** Histogram of the variable “Estimation of Duration” within the Floatation-REST condition. Area under the curve equals the total number of cases. The histogram shows the distribution of data. The estimate of the time was measured in minutes (x-axis) with the median of 60 minutes (grey bar) in the Floatation-REST condition.

**Figure S9:** Histogram of the variable “Estimation of Duration” within the Bed-REST condition. Area under the curve equals the total number of cases. The histogram shows the distribution of data. The estimate of the time was measured in minutes (x-axis) with the median of 49 minutes in the Bed-REST condition.
